# Impact of Dietary Supplementation with Sodium Butyrate Protected by Medium-Chain Fatty Acid Salts on Gut Health of Broiler Chickens

**DOI:** 10.3390/ani12192496

**Published:** 2022-09-20

**Authors:** Meritxell Sadurní, Ana Cristina Barroeta, Roser Sala, Cinta Sol, Mónica Puyalto, Lorena Castillejos

**Affiliations:** 1 Animal Nutrition and Welfare Service (SNiBA), Animal and Food Science Department, Faculty of Veterinary, Universitat Autònoma de Barcelona, 08193 Bellaterra, Spain; 2 Norel S.A., 28007 Madrid, Spain

**Keywords:** butyric acid, medium-chain fatty acid, feed additive, gut health, broiler

## Abstract

**Simple Summary:**

Nutritional strategies to improve gut health are under research to reduce antibiotic use in poultry production. This study investigated the effect of dietary supplementation with sodium butyrate protected by sodium salts of medium-chain fatty acids as a feed additive on broiler gut health. A first trial was conducted to assess the effectiveness of this feed additive supplemented at a dose range of 0.5, 1, and 2 kg/t to promote a good health status on broilers raised under optimal conditions. Supplementation at 0.5 and 1 kg/t maintained the number of mucin-secretory cells contained in the gut barrier of young chickens, and the use of 1 kg/t improved the intestinal immune system of aged broilers. However, the beneficial effects of some feed additives are not detected under non-challenged conditions. Therefore, the second experiment was performed to evaluate the effect of the feed additive at 1 kg/t in coccidiosis-challenged broilers. In this context, sodium butyrate protected by sodium salts of medium-chain fatty acids restored the number of mucin-secretory cells as well as impacted on the intestinal morphometry and microbiota. The results of the present study suggest that this feed additive could be a useful strategy to reinforce the gut barrier, especially for birds with coccidiosis.

**Abstract:**

Nutritional strategies to improve gut health of broilers are under research. This study investigated the effect of dietary supplementation with sodium butyrate protected by sodium salts of medium-chain fatty acids as a feed additive on broiler gut health. The first experiment was conducted to evaluate the effect of supplementing at 0.5, 1, and 2 kg/t in broilers housed under optimal conditions. Supplementation at 0.5 and 1 kg/t maintained goblet cell counts at 10 days of age (*p* ≤ 0.05), and supplementation at 1 kg/t decreased intraepithelial lymphocyte counts compared to 2 kg/t at 39 days (*p* ≤ 0.10). Abdominal fat pad levels of lauric and myristic acids were gradually increased by supplement dose (*p* ≤ 0.05). In the second experiment, the feed additive at 1 kg/t was evaluated in coccidiosis-challenged broilers. Experimental treatments were as follows: non-challenged, control-challenged, and supplemented-challenged treatments. Coccidiosis negatively impact performance and modify histomorphometry and microbiota (*p* ≤ 0.05). The feed additive increased crypt depth at 7 days post-inoculation and goblet cell count at 14 days post-inoculation (*p* ≤ 0.05). Further, supplementation interacted with the microbiota modification led by the coccidiosis (*p* ≤ 0.05). These results suggest that this feed additive could be a useful strategy to reinforce the gut barrier, especially for birds under coccidiosis-challenge treatments.

## 1. Introduction

Reduced use of antibiotics in poultry production has led to research designed to discover alternatives to improve gut health [1,2,3]. The intestinal tract is a vital organ for the digestion and absorption of nutrients, whereby a dynamic balance between its mucus layer and microbiota is essential for the barrier functions to maintain optimal bird performance and health [4].

Several studies have investigated the use of feed additives for broiler diets, focusing on natural sources [3,5]. Among the nutritional strategies tested, supplementing diets with short- and medium-chain fatty acids (SCFA; MCFA) has been found to help promote and maintain gut health in broilers chickens [4,6,7]. Butyric acid has been among the most widely investigated SCFA in poultry with improvements in feed conversion rate. It is a source of energy for intestinal cells that can positively influence their proliferation, differentiation, and maturation. Butyric acid also has antibacterial effect modulating the intestinal microbiota and positively affecting the intestinal barrier. This fatty acid (FA) is an essential natural product of microbial fermentation, although it is also available in synthetic form [8,9]. On the other hand, MCFA from coconut and palm kernel fatty acid distillates are byproducts of refining their corresponding crude vegetable oils and have also been a focus of research. Both SCFA and MCFA, and particularly butyric acid, are efficiently absorbed and metabolized in the gastrointestinal tract [7,10,11]. Therefore, the combination of salt forms may protect the fatty acids from immediate absorption and thus promote their beneficial effects throughout the intestinal tract [11,12]. The benefits of SCFA and MCFA additives for growth performance and gut health of broilers have not yet been confirmed [13,14]. Data suggest that these additives are most effective in animals such as broilers whose intestinal morphology or microbial balance is altered, compromising their intestinal integrity [13,15]. Avian coccidiosis is an infectious enteric disease caused by protozoan parasites of the genus *Eimeria* with a major impact in poultry production worldwide. Although coccidiosis may be subclinical, mucosal intestinal lesions can compromise performance parameters in broilers [16,17].

In this study, two experiments were conducted to examine the effect of dietary supplementation with the feed additive sodium butyrate protected by a mixture of sodium salts of MCFA on gut health of broiler chickens. The aim of the first experiment was to evaluate the effect of supplementing the diet at a dose range of 0.5 to 2 kg/t (0.5, 1, and 2 kg/t) in broilers housed under optimal conditions. In the second experiment, supplementation with sodium butyrate protected by sodium salts of medium-chain fatty acids at 1 kg/t was evaluated in broilers challenged with coccidiosis.

## 2. Materials and Methods

### 2.1. Animals, Housing, and Treatments

Both experiments were carried out at Servei de Granges i Camps Experimentals (Universitat Autònoma de Barcelona, Bellaterra, Barcelona, Spain). All experimental procedures were approved by the Animal Ethics Committee of this institution (Permit No. CEEAH 3938) and were in accordance with European Union Guidelines for the care and use of animals in research [18].

Newly hatched, female, Ross 308 broiler chickens obtained from a local hatchery (bonÀrea, Verdú, Lleida, Spain) were reared under controlled conditions of light and temperature, as recommended by the breeder. According to previous authors [19,20], females were used to evaluate the abdominal fat pat deposition and fatty acid composition.

***Experiment 1.*** A total of 192 chicks were randomly allocated to 24 cages housing 8 birds each. The design consisted of four treatments including a basal diet without supplementation (CTR) and the same basal diet with three doses of sodium butyrate protected by sodium salts of MCFA (DICOSAN+, Norel S.A., Madrid, Spain; DIC): 0.5 kg/t DIC (0.5DIC), 1 kg/t DIC (1DIC), and 2 kg/t DIC (2DIC). The DIC additive contains sodium butyrate at 70%, and 30% of sodium MCFA salts obtained from coconut fatty acid distillates (0.84% caprylic acid (C8:0), 0.84% capric acid (C10:0), 11.52% lauric acid (C12:0), 3.84% myristic acid (C14:0), 2.04% palmitic acid (C16:0), 0.60% stearic acid (C18:0), and 3.72% oleic acid (C18:1)).

***Experiment 2.*** For the coccidiosis challenge trial, 360 chicks were randomly allocated to 36 cages containing 10 animals each. The experimental treatments were as follows: non-challenged (NC), control-challenged (CC), and DIC supplemented-challenged treatments with 1 kg/t DIC (DC). On day 7, birds assigned to the challenged treatment groups received an attenuated vaccine (containing sporulated oocysts of *Eimeria* spp. (575 *Eimeria acervulina*, 345 *E. maxima*, 1150 *E. mitis*, and 575 *E. tenella*)) at 50× the recommended dose. The coccidial inoculum was introduced with a syringe (0.4 mL) in the oral cavity. Birds in the non-challenged group received a placebo. To facilitate the re-ingestion of oocysts during the trial period, the floor of each cage was lined with paper. To prevent cross contamination, non-challenged and challenged birds were kept in two separate identical rooms.

### 2.2. Diets

The feeding program was divided into two stages: chicks received a starter feed until day 21 and a grower-finisher feed from day 22 to 44. Diets were provided in mash form based on corn, wheat, and soybean meal formulated to meet or exceed FEDNA requirements [21] (Table 1*)*. Different amounts of DIC were included in a second mixture, which was used to replace the same amount of basal diet. Titanium dioxide was also added as non-digestible marker at 0.5% in Experiment 2. Throughout the study, feed and water were supplied ad libitum.

### 2.3. Controls, Sampling and Analytical Determinations

**Figure 1:**
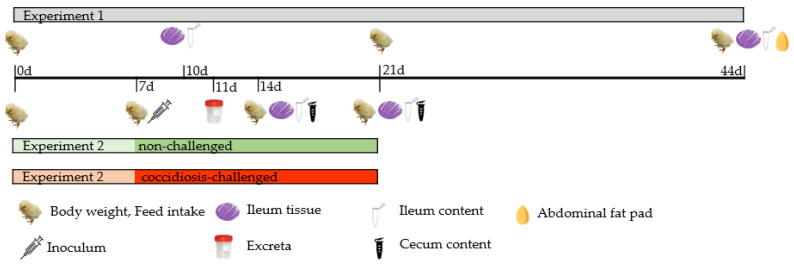
Sampling timeline. The figure shows the controls and sampling of Experiment 1 and Experiment 2.

The controls and samplings are shown in Figure 1. Experimental feed samples were taken at the beginning and the end of each experimental period, ground, and kept at 5 °C until further analyses. Proximate analysis of diets was performed according to AOAC International methods [22]: dry matter (Method 934.01), crude protein (Method 954.01), ether extract (Method 920.39), crude fiber (Method 962.09), and ash (Method 942.05). The GE was determined by adiabatic bomb calorimeter (IKA-Kalorimeter system C4000; Staufen, Germany). Fatty acid content was determined by the method of Sukhija and Palmquist [23] adding nonadecanoic acid (C19:0, Sigma-Aldrich Chemical Co.; St. Louis, MO, USA) as an internal standard for direct extraction-transesterification to prepare calibration curves and for FA quantification. The final extract obtained was injected into a gas chromatograph (HP6890, Agilent Technologies; Waldbronn, Germany) following the procedure described by Cortinas et al. [24]. To identify FA, their retention times were measured and compared with those of relative standards (Supelco 37 component FAME Mix, Sigma-Aldrich Co.; St. Louis, MO, USA) and contents quantified by internal normalization.

Broiler BW and feed intake were monitored at 21 and 44 days of age in Experiment 1, and at 7, 14, and 21 days in Experiment 2. These data were used to calculate the average daily feed intake (ADFI), average daily gain (ADG), and feed conversion ratio (FCR) for each period and for the overall study. Mortality was recorded daily to adjust ADFI and ADG.

**Figure 2:**
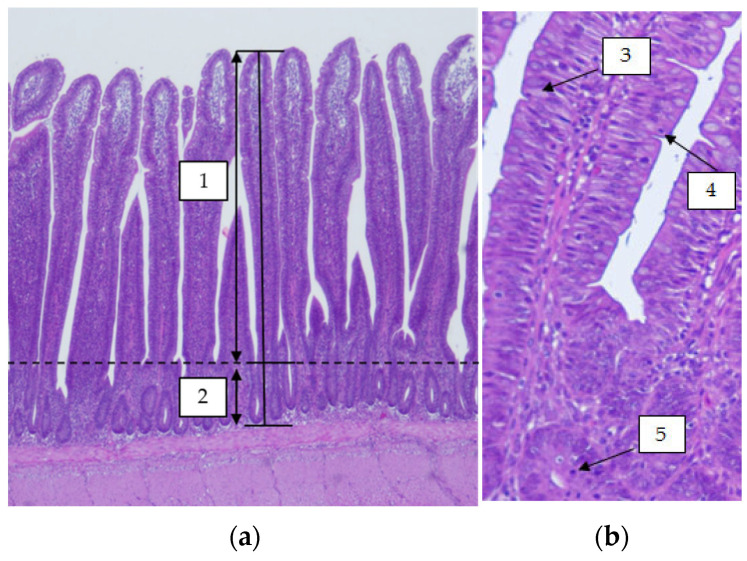
Histomorphometric measurements on ileum samples stained with hematoxylin and eosin. (**a**) Histological picture of 39-day-old broiler chickens’ ileum at 40× magnification. Villus height (1) and crypt depth (2). (**b**) Histological picture of 10-day-old broiler chicken at 100× magnification. Goblet cell (3), intraepithelial lymphocyte (4), and mitosis (5).

Ileum samples were collected during the experiments after the birds were stunned (electrical stunner Reference: 100523, FAF; Saint-Sernin-sur-Rance, France) and immediately exsanguinated. For the histological study, 2 cm long sections were removed from the middle of the ileum of 1 bird/replicate at 10 and 39 days of age in Experiment 1, and at 14 and 21 days in Experiment 2. Once harvested, the sections were partially opened longitudinally, washed thoroughly with sterile PBS, and fixed by immersion in a 4% formaldehyde solution. The tissue samples were then dehydrated and embedded in paraffin, sectioned at a thickness of 4 µm, and stained with hematoxylin and eosin. Morphometric variables (Figure 2) were analyzed using a light microscope (BHS, Olympus; Tokyo, Japan). Villus height (from the tip to an imaginary line connecting crypt top), crypt depth (from villus-crypt union to crypt base), and number of goblet cells and intraepithelial lymphocyte (IEL) in the villus and mitosis in the crypt were measured. Measurements were taken in well-oriented villus and crypts. The villus:crypt ratio was calculated by dividing villus height by crypt depth. The same villus and crypts columns were used to determine the number of goblet cells, IEL, and mitosis clearly distinguishable at 400× magnification [25].

Ileal contents on days 10 (5 birds/replicate) and 39 (1 bird/replicate) in Experiment 1, and on days 14 (4 birds/replicate) and 21 (3 birds/replicate) in Experiment 2, were collected and kept immediately on ice to calculate total lactic acid bacteria and enterobacterial counts. Ileal content samples were 6-fold serially diluted (1:10) in lactated Ringer’s solution (Sigma-Aldrich Co.; St. Louis, MO, USA) and used to inoculate MRS or MacConkey agar plates, which were incubated for 48 h. To quantitatively determine the total lactic acid bacteria, the culture plates were incubated in aerobic, microaerophilic (5% CO_2_), and anaerobic conditions at 37 °C. For enterobacterial counts, plates were incubated in aerobic conditions at 37 °C and 42 °C. Among all *Enterobacteriaceae*, coliforms and *Escherichia coli* were isolated in Experiment 1 and 2, respectively. In Experiment 2, *Clostridium perfringens* were counted in cecum contents on days 14 and 21 by inoculating diluted solutions in TSN agar at 45 °C until their solidification, followed by incubation in anaerobic conditions for 48 h at 37 °C. All counts are reported as colony-forming unit per gram (cfu/g).

In Experiment 1, abdominal fat pads (AFP) were individually collected and weighed at 44 days of age, and this weight was expressed as a percentage of live body weight. Collected AFP were then stored at −20 °C for fatty acid analysis according to the method of Carrapiso et al. [26]. The resultant extract was analyzed by gas chromatography following the method described above for the feed [24].

In Experiment 2, nutritional balance was assessed at day 11 of age, and excreta samples were taken (free of contaminants such as feed and feathers). The excreta samples were homogenized and stored at −20 °C until being freeze-dried, ground, and kept at 5 °C until further analyses. Excreta samples were analyzed by the same method as those described for feed to calculate AME. In addition, the apparent total tract digestibility (ATTD) of dry matter (DM), organic matter (OM), and FA were determined [22,23,24]. The inert marker was analyzed in feed and excreta following the procedures of Short et al. [27] and determined by ICP-OES spectrophotometry (Optima 3200 RL, Perkin Elmer; Waltham, MA, USA). Regarding the AME, it was determined from the product of the energy utilization ratio and its corresponding diet GE. Digestibility coefficients of DM, OM, and FA were determined using the TiO_2_ ratio in the diet and excreta according to the following equation:

Apparent digestibility of DM, OM, and FA = [1 − {([TiO_2_]_d_ × [N]_e_)/([TiO_2_]_e_ × [N]_d_)}] × 100 (1)

where [TiO_2_]_d_ and [N]_d_ are the concentration of the inert marker and the nutrient, in the diet, respectively, and [TiO_2_]_e_ and [N]_e_ are the concentration of each one of them in the excreta.

### 2.4. Statistical Analysis

Sample size was calculated using the package pwr.anova.test of R software version 3.6.0 with a significance level of 0.05 and 90% power of test. In Experiment 2, the sample size was increased due to the higher intragroup standard deviation that can be induced by coccidiosis challenge. 

All the results are expressed as means with their standard error of the mean. The effects of the experimental treatments on performance, intestinal parameters, digestibility parameters, and AFP composition were statistically analyzed also using the software R version 3.6.0. All analyses were performed using a linear model. Differences between means were tested using Tukey’s adjust correction for multiple comparisons. For microbial analysis, counts were log10-transformed and subjected to a Kruskal–Wallis test. When this test proved significant, pairwise combinations were contrasted with the Wilcoxon rank test. The experimental unit was the cage. Significance was set at *p* ≤ 0.05. A value of 0.05 < *p* ≤ 0.10 was considered a trend towards significance.

## 3. Results

### 3.1. Experiment 1

#### 3.1.1. Experimental Diet Characterization

The FA composition of the experimental diets is provided in Table 2. Most FA in the starter and grower-finisher diets were polyunsaturated fatty acids (PUFA), mostly linoleic acid (C18:2n6c), but also oleic and palmitic acids. The contents of MCFA (lauric acid) and myristic acid in the feed increased proportionally with the level of DIC supplementation.

#### 3.1.2. Performance Parameters

The trial was successfully carried out, and chickens showed good health throughout the study. The effects of DIC supplementation on growth performance in the starter (from 0 to 21 days) and grower-finisher (from 22 to 44 days) periods and overall study (from 0 to 44 days) are presented in Table 3. No statistically significant (*p* > 0.05) differences in performance parameters were detected between treatments in any period.

#### 3.1.3. Ileal Histomorphometry

The effects of the treatments on ileal histomorphology at 10 and 39days of age are shown in Table 4. No statistically significant (*p* > 0.05) differences were observed between the treatment groups in villus height and crypt depth. Cell counts were affected by DIC dose. Ten-day-old chicks receiving the additive DIC at 2 kg/t showed lower goblet cell counts than those given the lower DIC doses (*p* = 0.023). In 39-day-old broilers, the 2DIC treatment tended to give higher total IEL numbers than the 1DIC treatment (*p* = 0.085). Mitosis was not affected by the additive (*p* > 0.05).

#### 3.1.4. Microbiological Analysis

The effects of the treatments on the ileal microbiota are summarized in Table 5. No statistically significant (*p* > 0.05) differences were observed between the different DIC doses tested (0.5 kg/t to 2 kg/t) on the ileum microbiota of 10- or 39-day-old broilers. In 39-day-old broilers, the highest total lactic bacteria count was observed with the 1DIC treatment, whereas the 2DIC treatment gave the lowest count (*p* = 0.077).

#### 3.1.5. Abdominal Fat Pad Fatty Acid Composition

Effects of DIC supplementation on AFP percentages and its FA composition in 44-day-old birds are shown in Table 6. Levels of AFP, reported in grams and as a percentage of live body weight, were not changed by the experimental treatments (*p* > 0.05). The content of MCFA, particularly lauric acid (*p* < 0.001) and myristic acid (*p* < 0.001), increased progressively with the inclusion of higher levels of DIC.

### 3.2. Experiment 2

#### 3.2.1. Experimental Diet Characterization

The FA composition of the experimental treatments is provided in Table 7. Most FA in the starter diet were PUFA, mainly linoleic acid. Oleic and palmitic were also abundant in the diet. Lauric and myristic acids were only detected in the DC group.

#### 3.2.2. Performance Parameters

The effects of the treatments on performance parameters in the pre-inoculation (0 to 7 days of age) and post-inoculation (PI; 8 to 21 days of age) periods are shown in Table 8. Before birds received the coccidiosis-challenge treatment (0 to 7 days of age), no significant differences (*p* > 0.05) on performance parameters were observed between any of the treatments. The challenged broiler groups showed lower ADFI (*p* = 0.002) and ADG (*p* < 0.001) values from 8 to 14 days of age, and BW was lower at 14 days (*p* < 0.001) compared to the non-challenged group. However, over the period from 15 to 21 days of age, the challenged groups had higher ADFI and ADG than the non-challenged group (*p* < 0.001). Over the period of 0 to 21 days, the addition of 1 kg/t of DIC to the diet of coccidiosis-challenged broilers led to a higher ADFI (*p* = 0.017) and a trend toward a higher ADG (*p* = 0.060) compared to the NC group.

#### 3.2.3. Ileal Histomorphometry

Effects of treatments on ileal histomorphology on days 14 and 21 (7 and 14 days PI, respectively) are presented in Table 9. Villus height was not affected at either age, but crypt depth at 14 days of age was greater DC than NC (*p* = 0.019). Compared with the NC group, villus:crypt ratio tended to be lower in the DC group at day 14 (*p* = 0.075) and was lower in the CC group at day 21 (*p* = 0.048). No cell morphology differences were noted between treatments except for villus goblet cell count, which was lowest in CC group at 21 days of age (*p* = 0.001).

#### 3.2.4. Microbiological Analysis

Effects of treatments on ileal microbial counts at 14 and 21 days of age (7 and 14 days PI, respectively) are shown in Table 10. Total lactic acid bacteria count of NC birds were lower compared to the CC group at day 14 (*p* = 0.021) and lower compared to DC group at 21 days (*p* = 0.024). The total enterobacterial count at 14 days of age was lower in NC than the DC (*p* = 0.042). At 21 days, this count was higher in NC than CC (*p* = 0.048). The total lactic acid bacteria: *Enterobacteriaceae* ratio at 21 days of age was higher in the CC than NC group (*p* = 0.003). Among the *Enterobacteriaceae*, the *Escherichia coli* count at 21 days was also higher in NC than CC group (*p* = 0.042). No effects of the feed additive were noted on *Clostridium perfringens* counts from cecum contents.

#### 3.2.5. Digestibility Balance

The effects of treatments on dietary AME and digestibility at 11 days of age is shown in Table 11. The nutrient content (AME) and digestibility were similar between treatments in coccidiosis-challenged chickens regardless of dietary DIC supplementation.

## 4. Discussion

### 4.1. Experimental Diet Characterization

Feed supplementation with DIC was confirmed through FA characterization of the experimental diets. Accordingly, the detected concentrations of lauric and myristic acids in the supplemented groups of both experiments could be traced back to the coconut oil FA distillate byproduct included in the DIC [28].

### 4.2. Performance Parameters

Results from Experiment 1 and from the period prior to the coccidiosis challenge in Experiment 2 (days 0 to 7) revealed that DIC added to the diet at 0.5 kg/t to 2 kg/t had no effects on performance of healthy broilers housed under recommended conditions. Several authors have reported beneficial effects on performance from adding free and protected sodium butyrate or MCFA (0.2 to 3 kg/t) to feeds [7,8,11,29]. However, other authors [14,30] argue that the effects of SCFA or MCFA on performance of chicks under non-challenged conditions are often insignificant, as observed in the present study. Hence, according to Del Alamo et al. [15], the absence of differences in the first experiment under optimal health conditions led to compromise the intestinal integrity of broilers by coccidiosis-challenge treatment (Experiment 2) to show the beneficial effect of SCFA and MCFA.

The effectiveness of the coccidiosis-challenge model used in Experiment 2 was confirmed by significant reductions in ADFI and ADG (1 to 7 days PI) and BW at 7 days PI according to previous authors [17,31,32]. In contrast, during the recovery phase (8 to 14 days PI), performance was boosted, and higher ADFI and ADG values were recorded in the challenged groups. These results are not in agreement with other studies that have shown that performance parameters are impaired until 21 and 28 days PI [33,34]. However, the current challenge model involving inoculation of an oocyst mixture provided by a high dose of coccidiosis vaccine was able to recreate the impairment of performance parameters the first week PI seen in coccidial infections. 

Concerning DIC supplementation in the second experiment, it significantly increased ADFI and tended to improve ADG levels in challenged broilers compared to levels recorded in NC group. Previous authors observed higher ADG in coccidiosis-challenged broilers supplemented with butyrate glycerides at 4 kg/t or birds receiving sodium butyrate protected at 0.5 to 1 kg/t under necrotic enteritis (*Eimeria* and *Clostridium perfringens*) conditions [16,35,36]. In addition, Baltić et al. [7] summarized that in numerous studies MCFA seemed to show growth-promoting properties. Thus, significant increase in ADFI induced by DIC supplementation was considered the main reason for the tendency to improve growth performance in broilers according to Liu et al. [37]. Therefore, it could be considered that dietary supplementation with DIC, including butyric acid and medium-chain fatty acids, may be an effective nutritional strategy to increase feed intake and growth of broilers affected by coccidiosis.

### 4.3. Ileal Histomorphometry

In Experiment 1, increasing dietary levels of DIC up to 2 kg/t did not change villus height and crypt depth in 10- or 39-day-old healthy broilers. These observations agree with those of Khatibjoo et al. [38], who assessed sodium butyrate at 3 and 1.5 kg/t and MCFA at 1 kg/t, and their combination in a starter and finisher diet, respectively. In contrast, other authors noted increased villus height in the duodenum, jejunum and ileum in response to different forms of sodium butyrate and MCFA at similar doses (0.7 to 1.6 kg/t) to the ones used in the present study [7,39,40,41]. Thus, the results of Experiment 1 provide no data to suggest that butyric acid and MCFA improve gut health in broilers raised under optimal conditions.

In Experiment 2, the coccidiosis challenge reduced villus:crypt ratio at 14 and 21 days of age, in agreement with reported reductions in villus height and increased crypt depth caused by *Eimeria* infection [17,42]. On day 14, challenged broilers fed diets supplemented with DIC at 1 kg/t had deeper crypts than non-challenged broilers, suggesting rapid tissue turnover resulting in the tendency to have the lowest villus:crypt ratio. However, at 21 days of age, the reduced villus:crypt ratio induced by coccidiosis seemed to be offset by DIC supplementation, and a numerical increase in villus height was observed. These observations are consistent with Choct [43], who described crypt as the villus factory. Hence, it could be that DIC supplementation at 1 kg/t was able to stimulate villus growth as butyric acid is the main energy source for enterocytes [8]. Accordingly, the results of Experiment 2 support a significant role of butyric acid and MCFA in villus-crypt development in a damaged intestine. The result was an improvement in performance parameters for the overall study period.

In both experiments, goblet cell counts were affected by treatments. In Experiment 1, DIC supplementation at 2 kg/t reduced goblet cell counts compared with 0.5 or 1 kg/t DIC at 10 days of age. Consistently, Barcelo et al. [44] reported that high concentrations of butyrate may be toxic for goblet cells. In addition, previous authors observed an increase in goblet cells proliferation by supplementation with protected sodium butyrate at a dose range varying from 0.2 to 1 kg/t. However, the most effective doses were 0.8 and 1 kg/t [41,45]. Concerning MCFA, increased goblet cell counts by glycerol monolaurate at higher doses (3 and 5 kg/t) has been observed [3]. In this context, the supplementation with DIC at 1 kg/t was considered as the dose to evaluate in the second experiment with coccidial-challenged broilers. Therefore, sodium butyrate protected by MCFA at 1 kg/t prevented the reduction in goblet cells induced by coccidial infection at 21 days of age (14 days PI) suggesting that it may be a useful strategy to reinforce the intestinal mucosa.

On day 39 in Experiment 1, IEL counts tended to be lower following DIC supplementation at 1 kg/t than at the higher dose. This tendency for the 1DIC treatment to reduce IEL may be explained by butyrate’s anti-inflammatory effect, modulating intestinal immune cells such as lymphocytes, and a protective effect of MCFA downregulating local immune responses [8,46]. However, to the best of authors knowledge, no effects on the IEL counts have been observed in broilers supplemented with these fatty acids, while Decuypere and Dierick [47] reported an improvement of the activity of the immune system by MCFA, decreasing the IEL counts of piglets. Therefore, noting this tendency to reduce the number of IEL by 1 kg/t of the DIC additive was considered an interesting result to evaluate this dose in the second experiment under coccidial-challenged conditions. The lack of differences observed between treatments in Experiment 2 is inconsistent with reports by others who have described increased IEL numbers due to coccidiosis [48,49]. Thus, more studies are needed to examine the effects of butyric acid and MCFA on different intestinal segments in healthy and coccidiosis-challenged broilers.

Concerning the number of mitosis, no differences were observed in either experiment. In contrast, Onrust et al. [50] showed that butyrate induces intestinal crypt cell proliferation and, in parallel, decreases the apoptosis rate in the crypt increasing mitosis counts. Thus, while the number of cells undergoing mitosis was not affected in the present study, the observation of villus:crypt ratio changes in chicks with coccidiosis in the DIC-supplemented group suggests that butyric acid and MCFA could help reinforce histomorphometric parameters after the intestine has been damaged.

### 4.4. Microbiological Analysis

Higher total lactic acid bacteria count compared to *Enterobacteriaceae* were observed in Experiment 1, supporting the notion of Czerwinski et al. [39] that lactic acid bacteria dominate the commensal microbiota of the broiler intestine. Microbial populations in the gastrointestinal tract are determined by numerous factors [39,51]. Hence, the differences in lactic acid bacterial counts observed according to DIC dose in the ileum of 39-day-old broilers (Experiment 1) suggest a modulatory effect of butyric acid and MCFA on the intestinal microbiota. This tendency is supported by Thompson and Hinton [10], who reported that lactic acid bacteria is the microbial population most affected by FA. On the other hand, the coccidiosis performed in the Experiment 2 increasing total lactic bacteria count at 7 days PI and decreasing *Enterobacteriaceae* count at 14 days PI agrees with Baltić et al. [7] and Vieira et al. [52], who proposed that coccidiosis is a parasitic disease with effects on the diversity and composition of the intestinal microbial community.

Under coccidiosis-challenge conditions, DIC supplementation increased *Enterobacteriaceae* counts at 7 days PI compared to counts recorded in non-challenged broilers. This result did not confirm the results of others who reported a negative correlation between enterobacterial counts and the concentration of non-dissociated butyrate in broilers [39]. The contradictory result of the present study may be because coccidial infection interfered with antimicrobial effect of fatty acid. In addition, the increase in total lactic acid bacterial counts observed at 14 days PI in DIC-supplemented challenged broilers suggests that this is a strategy to inhibit pathogenic bacteria, providing an explanation for the lack of differences in *Enterobacteriaceae* counts observed. Increased lactic acid bacteria numbers could also contribute to increase broiler weight gain, as described by Vieira et al. [52].

In regard to *Clostridium perfringens* counts, although coccidiosis has been well documented as a predisposing factor for necrotic enteritis [17], no significant differences in clostridial counts were observed between treatments. This may be explained by the lower propensity of live attenuated anticoccidial vaccines to induce necrotic enteritis [53]. On the other hand, the present results agree with Kien et al. [54] and Timbermont et al. [55], who described no effects of butyric acid on *Clostridium perfringens*. However, inhibitory effects have been described of butyric acid in free and glyceride forms as well as in protected forms provided at doses of 0.2 to 7 kg/t [56,57]. In addition, according to Skřivanová et al. [58] and Timbermont et al. [55], lauric acid shows intense antimicrobial activity against *Clostridium perfringens*, supporting the idea that Gram-positive bacteria are more susceptible than Gram-negative bacteria to the inhibitory properties of MCFA. The results obtained do not support the notion that vaccine-induced coccidial infection exacerbates clostridial infections, despite the capacity observed of coccidiosis to modulate the microbial population in the ileum. Moreover, the antibacterial activity of butyric acid and MCFA against *Clostridium perfringens* could not be confirmed.

### 4.5. Abdominal Fat Pad Fatty Acid Composition

In Experiment 1, the use of up to 2 kg/t DIC was found to have no effect on relative percentage AFP. Several authors reported a reduction in fat tissues of broilers fed higher doses of butyrate or MCFA than the ones used here (2 to 6 kg/t) [59,60]. Hence, the DIC doses tested in the present trial may have been too low to promote any differences in percentage relative AFP.

According to various studies, the FA composition of AFP is a clear reflection of the dietary FA profile [19,20]. Here, a steady increase in AFP levels of lauric acid and myristic acid was detected with increasing doses of the additive. These results agree with Bach et al. [61], who concluded that the detection of MCFA in adipose tissue can be attributed to dietary supplementation. Therefore, DIC supplementation at 0.5, 1, and 2 kg/t seem sufficient for its detection in AFP but does not affect the amount of AFP.

### 4.6. Digestibility Balance

In Experiment 2, no differences in ATTD in terms of DM, OM, FA, and AME were detected in 11-day-old birds. These results disagree with previous authors who observed a reduction in nutrient digestibility, especially of fats and AME, in birds under coccidial infection. The effect reported by these authors depended on the type and number of *Eimeria* species administered in the challenge model and was significant in the specific intestinal region colonized by each species [62,63,64,65]. Hence, although ATTD were not affected in the present study, it is hypothesized that the *Eimeria maxima* from the vaccine inoculum may particularly effect jejunal or ileal digestibility because the middle small intestine is the main region parasitized by this species. In addition, Teng et al. [66] showed that intestinal permeability was lowest at 5 days PI using a higher dose of oocysts than in the present study, and at 6 days PI using a quite lower dose. Therefore, the present results support the argument made by these authors suggesting that 4 days PI may be too early to reveal a coccidial effect on digestibility.

Concerning DIC supplementation, no differences were detected on the supplemented broilers in the present study, although several authors observed improved nutrient digestibility and AME in birds supplemented with different forms of protected butyric acid and MCFA. In particular, the higher digestibility of crude fat and AME may be due to the improved secretion of pancreatic fluid induced by the butyrate salt [3,11,29,67]. Furthermore, Dibner and Buttin [68] suggested that acids and their salts improve digestibility balance by reducing microbial competition. However, no reduction in microbial population counts was observed at 7 days PI. Therefore, the present results invite further research on the effect of butyric acid and medium-chain fatty acids on digestibility.

## 5. Conclusions

Dietary supplementation with sodium butyrate protected by a mixture of sodium salts of MCFA gradually increase levels of lauric and myristic acids according to the doses, without modifying gut health. However, the supplementation at 0.5 and 1 kg/t reinforced the gut barrier, as reflected by higher goblet cell numbers compared to the use of the feed additive at 2 kg/t in healthy 10-day-old broiler chicks. In addition, at 39 days of age, the use of 1 kg/t of DIC appeared to lead to an anti-inflammatory effect that lowered IEL counts compared to counts obtained with 2 kg/t of DIC, along with an increased total lactic acid bacterial count. In this context, all the doses tested in Experiment 1 were able to modify the lipid profile of the AFP, gradually increasing levels of lauric and myristic acids.

Regarding the potential of DIC dietary supplementation under the coccidiosis challenge, the present model proved useful in providing a better understanding of the changes that took place. The greatest impact of this challenge was observed at around 7 days PI, when feed intake and weight gain were at the lowest. These results, in conjunction with the changes in microbiota and the increased crypt depth seen in the coccidiosis-challenged broilers supplemented with DIC, suggested that additional studies should be conducted to investigate its effect on nutrient digestibility one week PI. On the other hand, during the second week PI, higher feed intake and weight gain confirm a recovery phase of the challenged broilers that seems to be improved by DIC supplementation on the overall period of the present study. These results may be explained by the effects of DIC on improving intestinal gut barrier damaged by mixed *Eimeria* infection. Thus, the reductions produced in villus:crypt ratio and goblet cell numbers were attenuated by DIC supplementation, while lactic acid bacterial counts increased. In conclusion, dietary supplementation with sodium butyrate protected by a mixture of sodium salts of MCFA seems to be a useful nutrition strategy to reinforce the gut barrier, especially in broiler chickens challenged by a coccidial infection.

## Figures and Tables

**Table 1:** Ingredient composition and chemical analysis of the basal diets, as-fed basis (Experiment 1 and Experiment 2).

	Experiment 1		Experiment 2
	Starter Diet (From 0 to 21 days)	Grower-Finisher Diet (From 22 to 44 days)	Starter Diet (From 0 to 21 days)
Ingredients, %			
Corn	39.99	39.97	39.63
Wheat	24.46	30.76	24.46
Soybean meal 47%	28.97	22.90	28.97
Soybean oil	2.96	3.00	2.96
Bicalcium phosphate	1.14	0.69	1.14
Calcium carbonate	0.88	1.16	0.88
Salt	0.35	0.29	0.35
Premix ^1^	0.44	0.44	0.30
L-Lysine	0.34	0.28	0.34
DL-Methionine	0.31	0.23	0.31
L-Threonine	0.15	0.10	0.15
L-Valine	0.03	0.20	0.03
Sodium bicarbonate	0.05	0.04	0.05
Choline chloride	0.06	0.07	0.06
Titanium dioxide	-	-	0.50
Analyzed nutrient and energy content			
Dry matter, %	90.97	89.30	88.93
Crude protein, %	20.22	17.68	19.58
Ether extract, %	5.12	4.89	4.87
Crude fiber, %	1.83	2.66	2.72
Ash, %	5.82	4.58	6.33
Gross energy, kcal/kg	4072	4096	3978

^1^ Provided per kg feed: vitamin A (from retinol), 10,000 IU; vitamin D3 (from cholecalciferol), 2000 IU; vitamin E (from alfa-tocopherol), 15 mg; vitamin K3, 1.03 mg; vitamin B1, 0.12 mg; vitamin B2, 3.84 mg; vitamin B6, 1.20 mg; vitamin B12, 0.96 mg; calcium pantothenate, 10.58 mg; nicotinic acid, 25.20 mg; folic acid, 1.20 mg; Fe (from FeSO_4_), 24.84 mg; Mn (from MnSO_4_), 77.38 mg; Zn (from ZnO), 37.15 mg; Cu (from CuSO_4_), 8.10 mg; I (from KI) 0.84 mg; Se (from Na_2_SeO_3_).

**Table 2:** Fatty acid composition of the starter and grower-finisher diets ^1^ (Experiment 1).

	Starter Diet (From 0 to 21 days)				Grower-Finisher Diet (From 22 to 44 days)			
Fatty Acids, %	CTR	0.5DIC	1DIC	2DIC	CTR	0.5DIC	1DIC	2DIC
Saturated fatty acids	18.3	18.3	18.8	20.1	18.6	19.6	21.1	19.7
C12:0	-	0.19	0.42	0.87	-	0.21	0.40	0.63
C14:0	0.15	0.37	0.47	0.73	-	0.25	0.37	0.45
C16:0	14.1	13.8	13.9	14.4	14.6	15.1	16.0	14.6
C18:0	3.72	3.63	3.63	3.74	3.66	3.78	3.92	3.69
Monounsaturated fatty acids	25.3	25.3	25.0	25.9	25.3	25.9	26.2	25.0
C18:1n9c	23.7	23.8	23.3	23.9	24.0	24.8	24.9	23.7
Polyunsaturated fatty acids	56.4	56.3	56.2	54.0	56.1	54.5	52.7	55.3
C18:2n6c	51.1	51.2	50.9	49.0	51.2	50.0	48.1	50.5
C18:3n3	5.30	5.27	5.26	4.96	4.89	4.75	4.60	4.81
Minor fatty acids	2.02	2.02	2.13	2.41	1.66	1.67	1.65	1.65
UFA:SFA	4.46	4.46	4.32	3.97	4.37	4.09	3.74	4.07

^1^ Experimental treatments: CTR = basal diet; 0.5DIC = basal diet supplemented with 0.5 kg/t DIC; 1DIC = basal diet supplemented with 1 kg/t DIC; 2DIC = basal diet supplemented with 2 kg/t DIC. DIC = feed additive DICOSAN+; UFA:SFA = unsaturated-to-saturated fatty acid ratio.

**Table 3:** Effects of dietary DIC supplementation level on growth performance of broiler chickens (Experiment 1).

	Experimental Treatments ^1^				Statistics	
	CTR	0.5DIC	1DIC	2DIC	SEM	*p*-Value
From 0 to 21 d						
BW at 0 d, g	38.5	38.5	38.4	38.5	0.042	0.988
BW at 21 d, g	820	812	796	822	19.94	0.491
ADFI, g/d per bird	53.5	52.7	52.0	52.9	1.061	0.696
ADG, g/d per bird	37.0	36.6	35.9	36.9	0.910	0.547
FCR, g/g	1.45	1.44	1.44	1.43	0.022	0.960
From 22 to 44 d						
BW at 44 d, g	2663	2601	2588	2588	74.82	0.730
ADFI, g/d per bird	142	139	142	140	4.228	0.895
ADG, g/d per bird	80.5	77.6	77.2	76.2	2.343	0.508
FCR, g/g	1.82	1.79	1.85	1.84	0.053	0.818
From 0 to 44 d						
ADFI, g/d per bird	102	98.4	99.9	101	2.563	0.684
ADG, g/d per bird	59.7	58.4	58.0	57.9	1.703	0.730
FCR, g/g	1.70	1.69	1.72	1.71	0.042	0.913

^1^ CTR = basal diet; 0.5DIC = basal diet supplemented with 0.5 kg/t DIC; 1DIC = basal diet supplemented with 1 kg/t DIC; 2DIC = basal diet supplemented with 2 kg/t DIC. Values are means of 6 replicates. DIC = feed additive DICOSAN+; BW = body weight; ADFI = average daily feed intake; ADG = average daily gain; FCR = feed conversion ratio; SEM = standard error of the mean.

**Table 4:** Effects of dietary DIC supplementation level on histomorphological parameters recorded in the ileum of 10-day-old and 39-day-old broiler chickens (Experiment 1).

		Experimental Treatments ^1^				Statistics	
Ileum	Age	CTR	0.5DIC	1DIC	2DIC	SEM	*p*-Value
Villus							
Height, µm	10 d	352	356	371	368	20.70	0.778
	39 d	627	629	601	765	91.79	0.182
Goblet cells/villus	10 d	56.6 ^ab^	60.9 ^a^	60.2 ^a^	49.8 ^b^	3.861	0.023
	39 d	120	138	126	138	9.087	0.238
IEL/villus	10 d	4.72	5.82	5.55	5.53	0.812	0.558
	39 d	22.1 ^xy^	23.7 ^xy^	19.3 ^y^	28.5 ^x^	3.200	0.085
Crypt							
Depth, µm	10 d	160	164	167	158	11.41	0.854
	39 d	183	179	181	179	7.748	0.956
Mitosis/crypt	10 d	2.00	2.87	3.63	2.88	0.524	0.103
	39 d	1.08	1.73	1.25	1.99	0.523	0.303
Villus:crypt ratio							
	10 d	2.28	2.21	2.32	2.37	0.198	0.914
	39 d	3.48	3.57	3.39	4.37	0.598	0.187

^1^ CTR = basal diet; 0.5DIC = basal diet supplemented with 0.5 kg/t DIC; 1DIC = basal diet supplemented with 1 kg/t DIC; 2DIC = basal diet supplemented with 2 kg/t DIC. Values are means of 6 replicates. ^a–b^ Means within a row lacking a common superscript differ (*p* ≤ 0.05). ^x–y^ Means within a row lacking a common superscript differ (0.05 < *p* ≤ 0.10). DIC = feed additive DICOSAN+; IEL = intraepithelial lymphocytes; SEM = standard error of the mean.

**Table 5:** Effects of dietary DIC supplementation level on ileal microbiological counts in 10-day-old and 39-day-old broiler chickens (Experiment 1).

		Experimental Treatments ^1^				Statistics	
Ileum, Logcfu/g	Age	CTR	0.5DIC	1DIC	2DIC	SEM	*p*-Value
Total lactic acid bacteria	10 d	8.72	8.57	8.66	8.60	0.110	0.677
	39 d	8.95	8.96	8.99	8.90	0.041	0.077
*Enterobacteriaceae*	10 d	6.37	5.58	4.97	5.37	0.815	0.672
	39 d	3.68	3.57	3.82	4.30	0.849	0.798
Total lactic acid bacteria: *Enterobacteriaceae* ratio	10 d	1.50	1.67	1.85	1.65	0.208	0.691
	39 d	2.94	2.98	2.71	2.55	0.494	0.642
Total coliform bacteria	10 d	5.64	4.79	4.26	5.11	1.008	0.820
	39 d	3.53	2.91	3.67	3.81	0.747	0.686

^1^ CTR = basal diet; 0.5DIC = basal diet supplemented with 0.5 kg/t DIC; 1DIC = basal diet supplemented with 1 kg/t DIC; 2DIC = basal diet supplemented with 2 kg/t DIC. Values are means of 6 replicates. DIC = feed additive DICOSAN+; SEM = standard error of the mean.

**Table 6:** Effects of dietary DIC supplementation level on abdominal fat pat and its fatty acid composition in 44-day-old broiler chickens (Experiment 1).

	Experimental Treatments ^1^				Statistics	
Abdominal Fat Pat	CTR	0.5DIC	1DIC	2DIC	SEM	*p*-Value
g	48.6	38.9	40.4	42.0	3.567	0.220
% ^2^	1.83	1.48	1.55	1.62	0.128	0.190
Fatty acid profile, %						
Saturated fatty acids	29.4	29.3	29.5	29.1	0.655	0.958
C12:0	0.01 ^D^	0.06 ^C^	0.10 ^B^	0.16 ^A^	0.005	<0.001
C14:0	0.52 ^C^	0.54 ^BC^	0.58 ^AB^	0.62 ^A^	0.020	<0.001
C16:0	23.2	22.8	23.1	22.7	0.510	0.746
C18:0	5.36	5.59	5.40	5.35	0.179	0.646
C20:0	0.08	0.07	0.07	0.07	0.010	0.971
Monounsaturated fatty acids	44.7	44.6	45.5	45.4	0.737	0.499
C18:1n9c	36.5	36.7	37.5	37.3	0.518	0.250
Polyunsaturated fatty acids	25.9	26.1	25.0	25.5	0.654	0.553
C18:2n6c	23.0	23.2	22.2	22.7	0.570	0.544
C18:3n3	2.25	2.27	2.13	2.20	0.078	0.470
Minor fatty acids	9.18	8.81	8.92	8.98	0.324	0.738
UFA:SFA	2.40	2.51	2.37	2.38	0.068	0.240

^1^ CTR = basal diet; 0.5DIC = basal diet supplemented with 0.5 kg/t DIC; 1DIC = basal diet supplemented with 1 kg/t DIC; 2DIC = basal diet supplemented with 2 kg/t DIC. ^2^ Abdominal fat pad weight expressed as a percentage of live body weight. Values are means of 6 replicates. ^A–D^ Means within a row lacking a common superscript differ (*p* ≤ 0.01). DIC = feed additive DICOSAN+; UFA:SFA = unsaturated-to-saturated fatty acid ratio; SEM = standard error of the mean.

**Table 7:** Fatty acid compositions of the starter and grower-finisher diets ^1^ (Experiment 2).

	Starter Diet (From 0 to 21 days)		
Fatty Acids, %	NC	CC	DC
Saturated fatty acids	18.1	18.5	18.6
C12:0	-	-	0.29
C14:0	-	-	0.31
C16:0	16.0	16.3	15.9
C18:0	1.23	1.26	1.17
Monounsaturated fatty acids	29.5	29.8	29.5
C18:1n9c	27.7	28.0	27.7
Polyunsaturated fatty acids	52.4	51.7	52.0
C18:2n6c	48.4	47.9	48.0
C18:3n3	3.97	3.85	3.95
Minor fatty acids	2.69	2.71	2.68
UFA:SFA	4.53	4.40	4.38

^1^ Experimental treatments = NC = non-challenged; CC = control-challenged (coccidiosis); DC = DIC-supplemented-challenged (1 kg/t DIC). DIC = feed additive DICOSAN+; UFA:SFA = unsaturated-to-saturated fatty acid ratio.

**Table 8:** Effects of treatments on growth performance of broiler chickens challenged with coccidiosis (Experiment 2).

	Experimental Treatments ^1^			Statistics	
	NC	CC	DC	SEM	*p*-Value
From 0 to 7 d					
BW at 0 d, g	40.6	40.6	40.6	0.021	0.524
BW at 7 d, g	145	147	148	2.872	0.794
ADFI, g/d per bird	18.0	18.4	18.3	0.551	0.764
ADG, g/d per bird	15.0	15.2	15.3	0.409	0.787
FCR, g/g	1.20	1.21	1.21	0.030	0.959
From 8 to 14 d					
BW at 14 d, g	378 ^A^	351 ^B^	355 ^B^	5.600	<0.001
ADFI, g/d per bird	46.3 ^a^	41.5 ^b^	41.9 ^b^	1.206	0.002
ADG, g/d per bird	33.3 ^A^	28.5 ^B^	29.4 ^B^	0.523	<0.001
FCR, g/g	1.39	1.29	1.43	0.132	0.402
From 15 to 21 d					
BW at 21 d, g	687	700	720	12.19	0.124
ADFI, g/d per bird	67.6 ^B^	76.2 ^A^	80.5 ^A^	1.951	<0.001
ADG, g/d per bird	44.3 ^B^	50.2 ^A^	52.0 ^A^	1.592	<0.001
FCR, g/g	1.56	1.53	1.55	0.033	0.733
From 0 to 21 d					
ADFI, g/d per bird	43.8 ^b^	44.9 ^ab^	46.3 ^a^	0.637	0.017
ADG, g/d per bird	30.7 ^y^	31.2 ^xy^	32.3 ^x^	0.553	0.060
FCR, g/g	1.43	1.44	1.45	0.016	0.567

^1^ NC = non-challenged; CC = control-challenged (coccidiosis); DC = DIC-supplemented-challenged (1 kg/t DIC). Values are means of 12 replicates. ^A–B^ Means within a row lacking a common superscript differ (*p* ≤ 0.01). ^a–b^ Means within a row lacking a common superscript differ (*p* ≤ 0.05). ^x–y^ Means within a row lacking a common superscript differ (0.05 < *p* ≤ 0.10). DIC = feed additive DICOSAN+; BW = body weight; ADFI = average daily feed intake; ADG = average daily gain; FCR = feed conversion ratio; SEM = standard error of the mean.

**Table 9:** Effects of treatments on histomorphological ileal parameters at 7 and 14 days post-inoculation with the coccidial inoculum (Experiment 2).

		Experimental Treatments ^1^			Statistics	
	Age	NC	CC	DC	SEM	*p*-Value
Villus						
Height, µm	14 d	508	506	508	19.53	0.994
	21 d	637	582	619	22.34	0.103
Goblet cells/villus	14 d	80.7	85.7	86.5	4.621	0.498
	21 d	85.6 ^a^	71.0 ^b^	86.1 ^a^	3.159	0.001
IEL/villus	14 d	17.7	16.6	19.0	1.476	0.359
	21 d	16.6	14.5	17.5	1.209	0.129
Crypt						
Depth, µm	14 d	174 ^b^	182 ^ab^	196 ^a^	6.066	0.019
	21 d	148	159	164	5.598	0.114
Mitotic cells/crypt	14 d	1.35	1.37	1.36	0.193	0.995
	21 d	0.91	1.25	1.26	0.160	0.182
Villus:crypt ratio						
	14 d	2.98 ^x^	2.87 ^xy^	2.63 ^y^	0.118	0.075
	21 d	4.49 ^a^	3.79 ^b^	3.91 ^ab^	0.233	0.048

^1^ NC = non-challenged; CC = control-challenged (coccidiosis); DC = DIC-supplemented-challenged (1 kg/t DIC). Values are means of 12 replicates. A-B Means within a row lacking a common superscript differ (*p* ≤ 0.01). ^a–b^ Means within a row lacking a common superscript differ (*p* ≤ 0.05). ^x–y^ Means within a row lacking a common superscript differ (0.05 < *p* ≤ 0.10). DIC = feed additive DICOSAN+; IEL = intraepithelial lymphocytes; SEM = standard error of the mean.

**Table 10:** Effects of treatments on ileal and cecal microbial counts at 7 and 14 days post-inoculation with the coccidial inoculum (Experiment 2).

		Experimental Treatments ^1^			Statistics	
Logcfu/g	Age	NC	CC	DC	SEM	*p*-Value
Ileum						
Total lactic acid bacteria	14 d	6.91 ^b^	7.19 ^a^	7.18 ^ab^	0.100	0.021
	21 d	7.47 ^b^	8.32 ^ab^	8.43 ^a^	0.237	0.024
*Enterobacteriaceae*	14 d	7.37 ^b^	7.38 ^ab^	7.60 ^a^	0.217	0.042
	21 d	5.86 ^a^	4.80 ^b^	5.17 ^ab^	0.320	0.048
Total lactic acid bacteria:*Enterobacteriaceae* ratio	14 d	0.95	0.98	0.95	0.045	0.119
	21 d	1.32 ^b^	1.80 ^a^	1.66 ^ab^	0.102	0.003
*Escherichica coli*	14 d	5.05	5.73	5.90	0.315	0.116
	21 d	5.80 ^a^	4.68 ^b^	4.86 ^ab^	0.326	0.042
Cecum						
*Clostridium perfringens*	14 d	2.91	3.06	4.20	0.681	0.178
	21 d	2.73	2.74	1.85	0.685	0.620

^1^ NC = non-challenged; CC = control-challenged (coccidiosis); DC = DIC-supplemented-challenged (1 kg/t DIC). Values are means of 12 replicates. ^a–b^ Means within a row lacking a common superscript differ (*p* ≤ 0.05). DIC = feed additive DICOSAN+; SEM = standard error of the mean.

**Table 11:** Effects of treatments on dietary AME (kcal/kg) along with dry matter, organic matter, and fatty acid digestibility (%) in 11-day-old broiler chickens (Experiment 2).

	Experimental Treatments ^1^			Statistics	
	NC	CC	DC	SEM	*p*-Value
AME, kcal/kg	3138	3082	3180	39.78	0.361
Digestibility, %					
Dry matter	90.65	90.35	90.42	0.384	0. 835
Organic matter	70.09	68.89	70.26	0.981	0.375
Total FA	73.97	72.54	75.74	2.305	0.517
SFA	60.87	58.14	62.73	3.523	0.531
MUFA	72.34	70.91	74.36	2.707	0.557
PUFA	77.74	76.64	79.46	1.958	0.494

^1^ NC = non-challenged; CC = control-challenged (coccidiosis); DC = DIC-supplemented-challenged (1 kg/t DIC). Values are means of 6 replicates. DIC = feed additive DICOSAN+; AME = apparent metabolizable energy; FA = fatty acids; SFA = saturated fatty acids; MUFA = monounsaturated fatty acids; PUFA = polyunsaturated fatty acids; UFA = unsaturated fatty acids; SEM = standard error of the mean.

## Data Availability

The data presented in this study are available on request from the corresponding author.
